# High-resolution contrast-enhanced MRI with three-dimensional fast spin echo improved the diagnostic performance for identifying pituitary microadenomas in Cushing’s syndrome

**DOI:** 10.1007/s00330-023-09585-1

**Published:** 2023-05-22

**Authors:** Zeyu Liu, Bo Hou, Hui You, Lin Lu, Lian Duan, Mingli Li, Kan Deng, Yong Yao, Huijuan Zhu, Feng Feng

**Affiliations:** 1 Department of Radiology, Peking Union Medical College Hospital, Chinese Academy of Medical Sciences and Peking Union Medical College, No. 1 Shuaifuyuan Wangfujing Dongcheng Distinct, Beijing, 100730 China; 2 Department of Endocrinology, Peking Union Medical College Hospital, Chinese Academy of Medical Sciences and Peking Union Medical College, No. 1 Shuaifuyuan Wangfujing Dongcheng Distinct, Beijing, 100730 China; 3 Department of Neurosurgery, Peking Union Medical College Hospital, Chinese Academy of Medical Sciences and Peking Union Medical College, No. 1 Shuaifuyuan Wangfujing Dongcheng Distinct, Beijing, 100730 China; 4 State Key Laboratory of Complex Severe and Rare Disease, Peking Union Medical College Hospital, Chinese Academy of Medical Sciences and Peking Union Medical College, No. 1 Shuaifuyuan Wangfujing Dongcheng Distinct, Beijing, 100730 China

**Keywords:** Magnetic resonance imaging, Spin echo imaging, Diagnosis, ACTH-secreting pituitary adenomas, Cushing’s syndrome

## Abstract

**Objectives:**

To assess the diagnostic performance of high-resolution contrast-enhanced MRI (hrMRI) with three-dimensional (3D) fast spin echo (FSE) sequence by comparison with conventional contrast-enhanced MRI (cMRI) and dynamic contrast-enhanced MRI (dMRI) with 2D FSE sequence for identifying pituitary microadenomas.

**Methods:**

This single-institutional retrospective study included 69 consecutive patients with Cushing’s syndrome who underwent preoperative pituitary MRI, including cMRI, dMRI, and hrMRI, between January 2016 to December 2020. Reference standards were established by using all available imaging, clinical, surgical, and pathological resources. The diagnostic performance of cMRI, dMRI, and hrMRI for identifying pituitary microadenomas was independently evaluated by two experienced neuroradiologists. The area under the receiver operating characteristics curves (AUCs) were compared between protocols for each reader by using the DeLong test to assess the diagnostic performance for identifying pituitary microadenomas. The inter-observer agreement was assessed by using the *κ* analysis.

**Results:**

The diagnostic performance of hrMRI (AUC, 0.95–0.97) was higher than cMRI (AUC, 0.74–0.75; *p* ≤ .002) and dMRI (AUC, 0.59–0.68; *p* ≤ .001) for identifying pituitary microadenomas. The sensitivity and specificity of hrMRI were 90–93% and 100%, respectively. There were 78% (18/23) to 82% (14/17) of the patients, who were misdiagnosed on cMRI and dMRI and correctly diagnosed on hrMRI. The inter-observer agreement for identifying pituitary microadenomas was moderate on cMRI (*κ* = 0.50), moderate on dMRI (*κ* = 0.57), and almost perfect on hrMRI (*κ* = 0.91), respectively.

**Conclusions:**

The hrMRI showed higher diagnostic performance than cMRI and dMRI for identifying pituitary microadenomas in patients with Cushing’s syndrome.

**Key Points:**

• *The diagnostic performance of hrMRI was higher than cMRI and dMRI for identifying pituitary microadenomas in Cushing’s syndrome.*

• *About 80% of patients, who were misdiagnosed on cMRI and dMRI, were correctly diagnosed on hrMRI.*

• *The inter-observer agreement for identifying pituitary microadenomas was almost perfect on hrMRI.*

**Supplementary Information:**

The online version contains supplementary material available at 10.1007/s00330-023-09585-1.

## Introduction

Cushing’s syndrome, caused by excessive exposure to glucocorticoids, is associated with considerable morbidity and increased mortality [1]. Cushing’s syndrome has diverse manifestations, including central obesity, moon facies, purple striae, and hypertension [2]. Cushing’s disease, due to adrenocorticotropic hormone (ACTH) hypersecretion from pituitary adenomas, is the most common etiology of ACTH-dependent Cushing’s syndrome [1, 2]. According to the Endocrine Society Clinical Practice Guideline, transsphenoidal surgery is the first-line treatment for Cushing’s disease [3]. The identification of pituitary adenomas on preoperative MRI can significantly increase the postoperative remission rate from 50 to 98% [4]. Therefore, it is critical to identify pituitary adenomas on MRI before surgery.

However, there are considerable challenges in identifying ACTH-secreting pituitary adenomas. This is because about 90% of the tumors are microadenomas (less than 10 mm in size) and the median diameter at surgery is about 5 mm [5, 6]. Conventional contrast-enhanced MRI (cMRI) using a two-dimensional (2D) fast spin echo (FSE) sequence has been routinely used to acquire images with 2- to 3-mm slice thickness, but some microadenomas are difficult to be identified on cMRI, resulting in false negatives reported in up to 50% of patients with Cushing’s disease [7]. Dynamic contrast-enhanced MRI (dMRI) increases the sensitivity of identifying pituitary adenomas to 66% [8], but it also increases false positives at the same time [9, 10]. The 3D spoiled gradient recalled (SPGR) sequence has been introduced in high-resolution contrast-enhanced MRI (hrMRI) to acquire images with 1- to 1.2-mm slice thickness. It is reported that the 3D SPGR sequence is superior to the 2D FSE sequence in the identification of pituitary adenomas with a sensitivity of up to 80% [11–13], but it cannot satisfy the clinical needs that about 20% of the lesions are still missed. Therefore, techniques are needed that can help better identify pituitary adenomas, particularly microadenomas. Previously, the 3D FSE sequence was recommended in patients with hyperprolactinemia [14]. Recently, the 3D FSE sequence has developed rapidly and can provide superior image quality with diminished artifacts [15]. Sartoretti et al demonstrated in a very effective fashion that the 3D FSE sequence is a reliable alternative for pituitary imaging in terms of image quality [16]. However, to our knowledge, few studies have investigated the diagnostic performance of 3D FSE sequences for identifying ACTH-secreting pituitary adenomas, particularly microadenomas.

The aim of our study was to assess the diagnostic performance of hrMRI with 3D FSE sequence by comparison with cMRI and dMRI with 2D FSE sequence for identifying ACTH-secreting pituitary microadenomas in patients with Cushing’s syndrome.

## Materials and methods

This single-institutional retrospective study was approved by the Institutional Review Board of our hospital. The study was conducted in accordance with the Helsinki Declaration. The informed consent was waived due to the retrospective nature of the study.

### Study participants

**Fig. 1:**
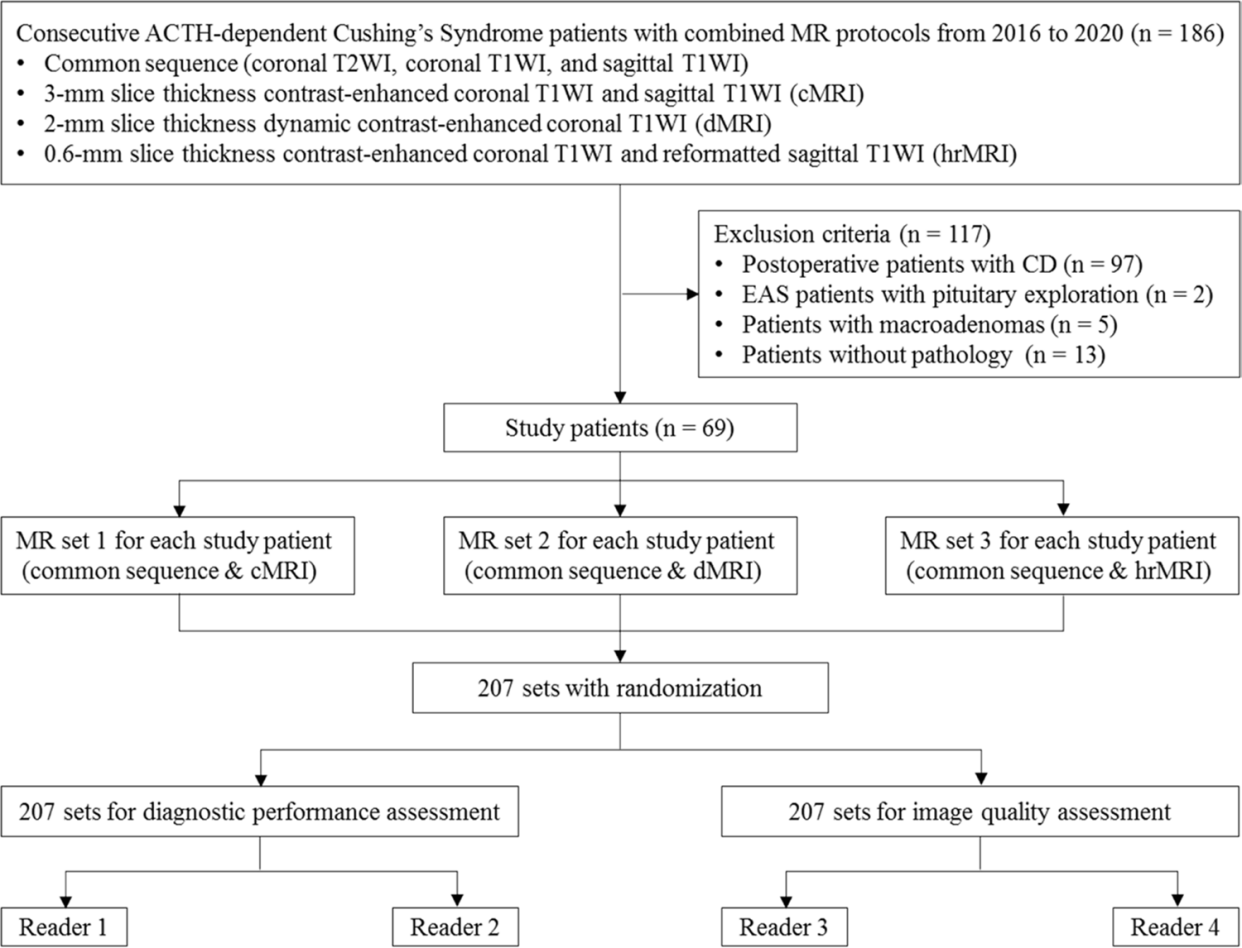
Flowchart of patient inclusion/exclusion process and image analysis. ACTH adrenocorticotropic hormone, CD Cushing’s disease, EAS ectopic ACTH syndrome, T1WI T1-weighted imaging, T2WI T2-weighted imaging

We retrospectively reviewed the medical records and imaging studies of 186 consecutive patients with ACTH-dependent Cushing’s syndrome, who underwent a combined protocol of cMRI, dMRI, and hrMRI from January 2016 to December 2020. Postoperative patients with Cushing’s disease (*n* = 97), patients with ectopic ACTH syndrome who underwent pituitary exploration (*n* = 2), and patients with macroadenomas (*n* = 5) or lack of pathology (*n* = 13) were excluded from the study. Finally, 69 patients with ACTH-dependent Cushing’s syndrome were included in the current study (Fig. 1) and the patients included were all surgically confirmed.

### MRI protocol

All the patients were imaged on a 3.0 Tesla MR scanner (Discovery MR750w, GE Healthcare) using an 8-channel head coil. The MRI protocol included coronal T2-weighted imaging, coronal T1-weighted imaging, and sagittal T1-weighted imaging before contrast injection. After contrast injection of gadopentetate dimeglumine (Gd-DTPA) at 0.05 mmol/kg (0.1 mL/kg) with a flow rate of 2 mL/s followed by a 10-mL saline solution flush, dMRI and cMRI with 2D FSE sequence were obtained first, and hrMRI with 3D FSE sequence using variable flip angle technique was performed immediately afterward. Detailed acquisition parameters are presented in Table S1.

### Image analysis: diagnostic performance

Image interpretation was independently conducted by two experienced neuroradiologists (F.F. and H.Y. with 25 and 16 years of experience in neuroradiology, respectively), who were blinded to patient information. The evaluation order of cMRI, dMRI, and hrMRI sequences was randomized. The identification of pituitary microadenomas on images was scored based on a three-point scale (0 = poor; 1 = fair; 2 = excellent). Scores of 1 or 2 represented the identification of the lesion. Reference standards were established by using all available imaging, clinical, surgical, and pathological resources, with a multidisciplinary team approach.

### Image analysis: image quality

Two readers (Z.L. and B.H. with 4 years of experience in radiology, respectively) were asked to assess the image quality of cMRI, dMRI, and hrMRI. Before exposure to images used in the current study, these readers underwent a training session to make sure that they were comparable to the experienced neuroradiologists in terms of image quality assessment. Images were presented in a random order. Image quality was assessed by using a 5-point Likert scale [17], including overall image quality (1 = non-diagnostic; 2 = poor; 3 = fair; 4 = good; 5 = excellent), sharpness (1 = non-diagnostic; 2 = not sharp; 3 = a little sharp; 4 = moderately sharp; 5 = satisfyingly sharp), and structural conspicuity (1 = non-diagnostic; 2 = poor; 3 = fair; 4 = good; 5 = excellent). An example of image quality assessment is shown in Table S2. Final decision was made through a consensus agreement.

The mean signal intensity of pituitary microadenomas, pituitary gland, and noise on cMRI, dMRI, and hrMRI was measured using an operator-defined region of interest. For noise, a 10-mm^2^ region of interest was placed in the background, and noise was defined as the standard deviation of the signal intensity of the background [17]. For pituitary microadenomas and pituitary gland, the region of interest should include a representative portion of the structure. The mean signal intensity of the pituitary microadenoma was replaced with that of the pituitary gland when no microadenoma was identified. A signal-to-noise ratio (SNR) was defined as the mean signal intensity of the pituitary microadenoma divided by noise. A contrast-to-noise ratio (CNR) was defined as the absolute difference of the mean signal intensity between the normal pituitary gland and pituitary microadenomas divided by noise [17]. Supplementary Fig. 1 shows how to measure the SNR and CNR with the region of interest in a contrast-enhanced pituitary MRI. Supplementary Fig. 2 shows the selection of images for the SNR and CNR calculation.

### Statistical analysis

The *κ* analysis was conducted to assess the inter-observer agreement for identifying pituitary microadenomas. The *κ* value was interpreted as follows: below 0.20, slight agreement; 0.21–0.40, fair agreement; 0.41–0.60, moderate agreement; 0.61–0.80, substantial agreement; greater than 0.80, almost perfect agreement.

To assess the diagnostic performance of cMRI, dMRI, and hrMRI for identifying pituitary microadenomas, the receiver operating characteristic curves were plotted and the area under curves (AUCs) were compared between MR protocols for each reader by using the DeLong test. Sensitivity, specificity, positive predictive value, and negative predictive value were calculated. The Mann–Whitney *U* test was used to evaluate the difference in image quality scores and the Wilcoxon signed-rank test was used to evaluate SNR and CNR measurements between MR protocols. A *p* value of less than 0.05 was considered statistically significant. Statistical analysis was performed using MedCalc Statistical Software (version 20.0.15; MedCalc Software) and SPSS Statistics (version 22.0; IBM).

## Results

### Clinical characteristics

A total of 69 patients (median age, 39 years; interquartile range [IQR], 29–54 years; 38 women [55%]) with ACTH-dependent Cushing’s syndrome were included in the study and their clinical characteristics are shown in Table 1. Among the 69 patients, 60 (87%) patients were diagnosed with Cushing’s disease and 9 (13%) were ectopic ACTH syndrome. The median disease course was 36 months (IQR, 12–78 months). The median serum cortisol, ACTH, and 24-h urine free cortisol level before surgery were 33.0 μg/dL (IQR, 25.1–40.1 μg/dL; normal range 4.0–22.3 μg/dL), 77.2 ng/L (IQR, 55.0–124.0 ng/L; normal range 0–46 ng/L), and 422.0 μg (IQR, 325.8–984.6 μg; normal range 12.3–103.5 μg), respectively. The median serum cortisol and 24-h urine free cortisol level after surgery were 3.0 μg/dL (IQR, 1.8–18.4 μg/dL) and 195.6 μg (IQR, 63.5–1240.3 μg), respectively. The median diameter of pituitary microadenomas was 5 mm (IQR, 4–5 mm), ranging from 3 to 9 mm.

**Table 1:** Clinical characteristics of the patients

Characteristics	Value
No. of patients	69
No. of female patients*	38 (55)
Age (years)	39 (29–54)
Disease course (months)	36 (12–78)
Size of pituitary microadenomas (mm)	5 (4–5)
Preoperative cortisol (μg/dL)	33.0 (25.1–40.1)
Preoperative ACTH (ng/L)	77.2 (55.0–124.0)
Preoperative 24hUFC (μg)	422.0 (325.8–984.6)
Postoperative cortisol (μg/dL)	3.0 (1.8–18.4)
Postoperative 24hUFC (μg)	195.6 (63.5–1240.3)

Except where indicated, data are medians with interquartile ranges in parentheses Abbreviations: *ACTH*, adrenocorticotropic hormone; *24hUFC*, 24-h urine free cortisol

^*^Data are numbers of patients, with percentages in parentheses

### Diagnostic performance of cMRI, dMRI, and hrMRI for identifying pituitary microadenomas

The inter-observer agreement for identifying pituitary microadenomas by *κ* statistic between two readers was moderate on cMRI (*κ* = 0.50), moderate on dMRI (*κ* = 0.57), and almost perfect on hrMRI (*κ* = 0.91), respectively.

The diagnostic performance for identifying pituitary microadenomas on cMRI, dMRI, hrMRI, and combined cMRI and dMRI is summarized in Table 2. For reader 1, the diagnostic performance of hrMRI (AUC, 0.95; 95%CI: 0.87, 0.99) was higher than that of cMRI (AUC, 0.75; 95%CI: 0.63, 0.85; *p* = 0.002), dMRI (AUC, 0.59; 95%CI: 0.47, 0.71; *p* < 0.001), and combined cMRI and dMRI (AUC, 0.65; 95%CI: 0.53, 0.76; *p* = 0.001). For reader 2, the diagnostic performance of hrMRI (AUC, 0.97; 95%CI: 0.89, 1.00) was higher than that of cMRI (AUC, 0.74; 95%CI: 0.63, 0.84; *p* = 0.001), dMRI (AUC, 0.68; 95%CI: 0.56, 0.79; *p* = 0.001), and combined cMRI and dMRI (AUC, 0.70; 95%CI: 0.58, 0.80; *p* = 0.003).

**Table 2:** Diagnostic performance of cMRI, dMRI, and hrMRI for identifying pituitary microadenomas

	AUC	*p* value	Sensitivity (%)	Specificity (%)	PPV (%)	NPV (%)
Reader 1						
hrMRI	0.95 (0.87, 0.99)		90 (80, 96) [54/60]	100 (66, 100) [9/9]	100 (93, 100) [54/54]	60 (32, 84) [9/15]
cMRI	0.75 (0.63, 0.85)	.002*	62 (48, 74) [37/60]	89 (52, 100) [8/9]	97 (86, 100) [37/38]	26 (12, 45) [8/31]
dMRI	0.59 (0.47, 0.71)	< .001^†^	52 (38, 65) [31/60]	67 (30, 93) [6/9]	91 (76, 98) [31/34]	17 (7, 34) [6/35]
cdMRI	0.65 (0.53, 0.76)	.001^‡^	63 (50, 75) [38/60]	67 (30, 93) [6/9]	93 (80, 99) [38/41]	21 (8, 41) [6/28]
Reader 2						
hrMRI	0.97 (0.89, 1.00)		93 (84, 98) [56/60]	100 (66, 100) [9/9]	100 (94, 100) [56/56]	69 (39, 91) [9/13]
cMRI	0.74 (0.63, 0.84)	.001*	60 (47, 72) [36/60]	89 (52, 100) [8/9]	97 (86, 100) [36/37]	25 (12, 43) [8/32]
dMRI	0.68 (0.56, 0.79)	.001^†^	70 (57, 81) [42/60]	67 (30, 93) [6/9]	93 (82, 99) [42/45]	25 (10, 47) [6/24]
cdMRI	0.70 (0.58, 0.80)	.003^‡^	73 (60, 84) [44/60]	67 (30, 93) [6/9]	94 (83, 99) [44/47]	27 (11, 50) [6/22]

Data in parentheses are 95% confidence intervals; data in brackets are raw data. Abbreviations: *AUC*, area under the receiver operating characteristic curve; *cdMRI*, combined cMRI and dMRI; *cMRI*, conventional contrast-enhanced MRI; *dMRI*, dynamic enhanced MRI; *hrMRI*, high-resolution contrast-enhanced MRI; *NPV*, negative predictive value; *PPV*, positive predictive value

^*^p value for comparison of the AUC between cMRI and hrMRI

^†^*p* value for comparison of the AUC between dMRI and hrMRI

^‡^*p* value for comparison of the AUC between cdMRI and hrMRI

For reader 1, 23 of the 69 patients (33%) were misdiagnosed on both cMRI and dMRI, but 18 of the 23 misdiagnosed patients (78%) were correctly diagnosed on hrMRI. For reader 2, 17 of the 69 patients (25%) were misdiagnosed on both cMRI and dMRI, but 14 of the 17 misdiagnosed patients (82%) were correctly diagnosed on hrMRI.

**Fig. 2:**
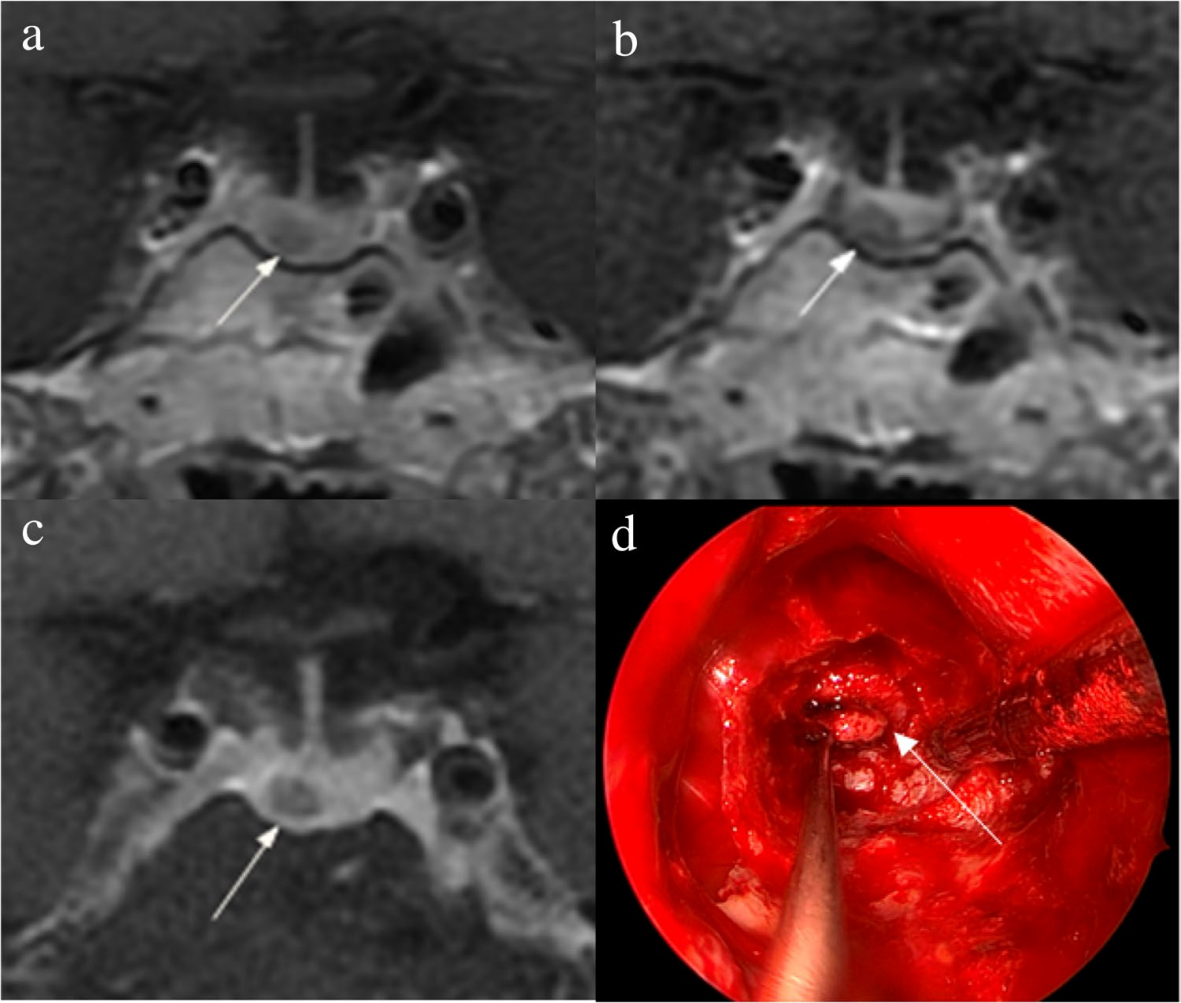
Images in a 56-year-old man with Cushing’s disease. The 5-mm pituitary microadenoma (arrow) can be identified on (**a**) coronal contrast-enhanced T1-weighted image and (**b**) coronal dynamic contrast-enhanced T1-weighted image obtained with two-dimensional (2D) fast spin echo (FSE) sequence, but the margin is not fully delineated. The lesion (arrow) is well delineated on (**c**) coronal contrast-enhanced T1-weighted image on high-resolution MRI obtained with 3D FSE sequence. **d** Intraoperative endoscopic photograph during transsphenoidal surgery after exposure of the sellar floor shows a round pituitary microadenoma (arrow)

**Fig. 3:**
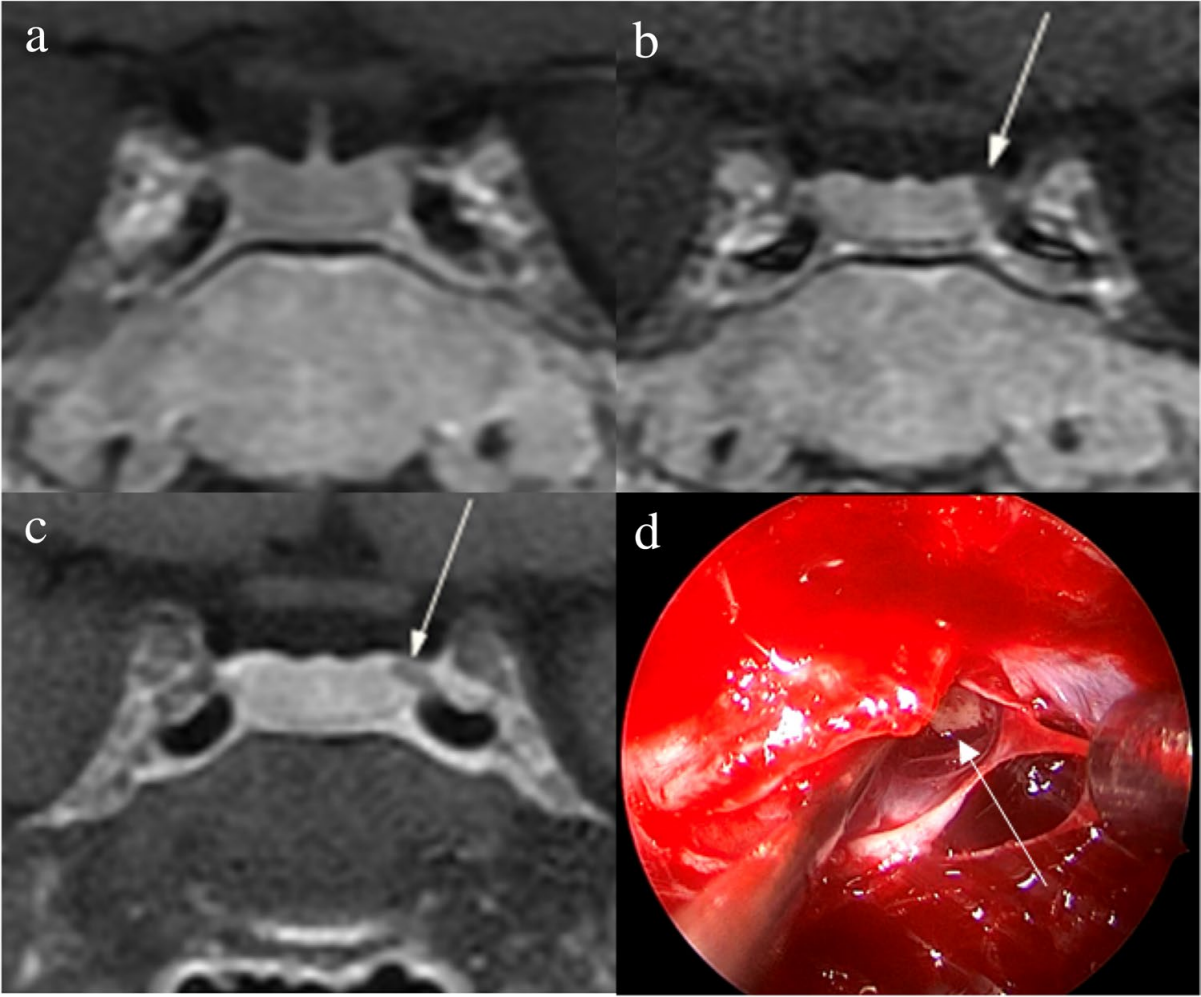
Images in a 34-year-old woman with Cushing’s disease. No tumor is identified on (**a**) coronal contrast-enhanced T1-weighted image obtained with two-dimensional (2D) fast spin echo (FSE) sequence. The 3-mm pituitary microadenoma (arrow) with delayed enhancement is identified on the left side of the pituitary gland on (**b**) coronal dynamic contrast-enhanced T1-weighted image obtained with 2D FSE sequence and (**c**) coronal contrast-enhanced T1-weighted image on high-resolution MRI obtained with 3D FSE sequence. **d** Intraoperative endoscopic photograph during transsphenoidal surgery shows a 3-mm pituitary microadenoma (arrow)

**Fig. 4:**
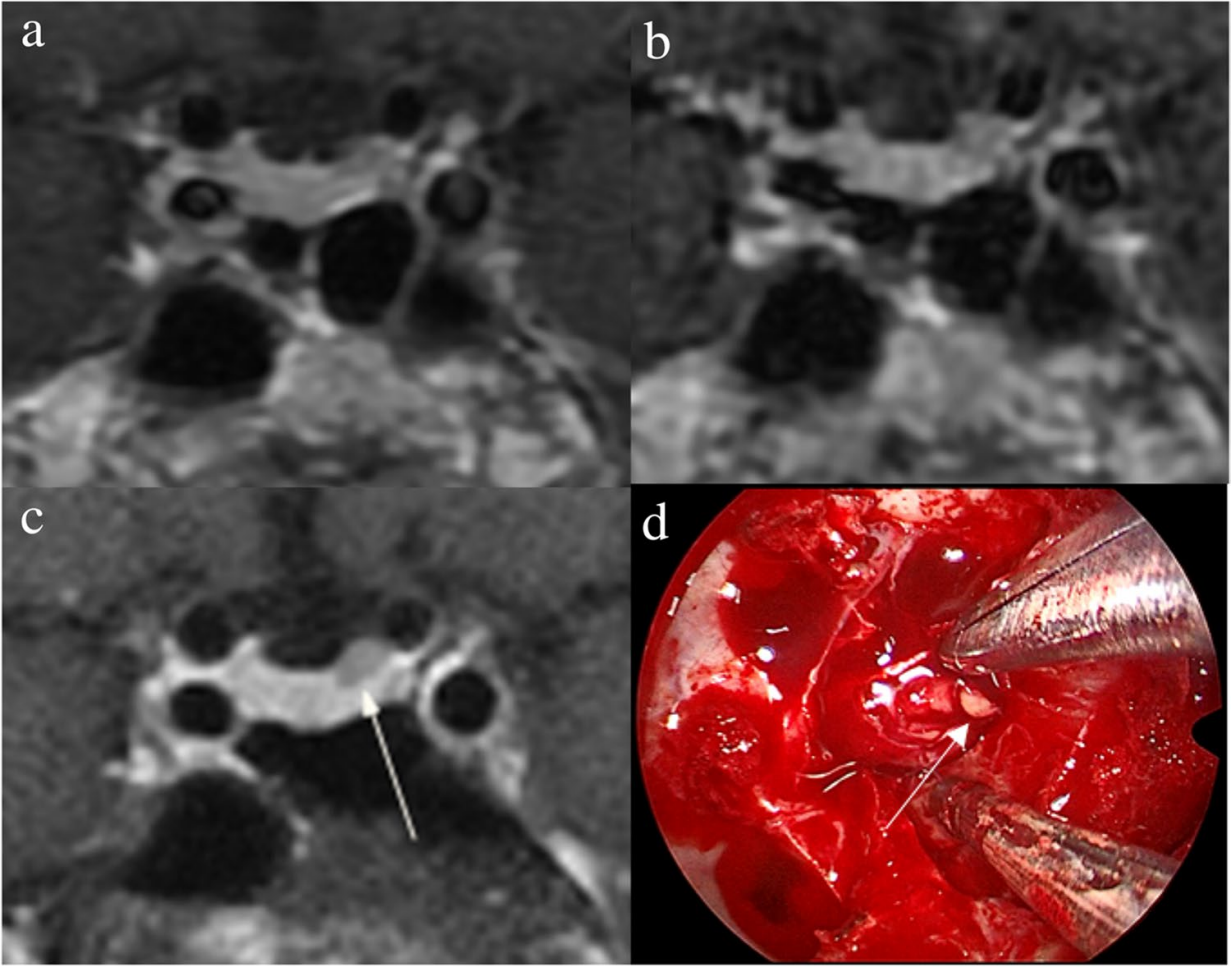
Images in a 43-year-old man with Cushing’s disease. The lesion is missed on (**a**) coronal contrast-enhanced T1-weighted image and (**b**) coronal dynamic contrast-enhanced T1-weighted image obtained with two-dimensional (2D) fast spin echo (FSE) sequence. **c** Coronal contrast-enhanced T1-weighted image on high-resolution MRI obtained with 3D FSE sequence shows a round pituitary microadenoma (arrow) measuring approximately 5 mm with delayed enhancement on the left side of the pituitary gland. **d** Intraoperative endoscopic photograph for microsurgical resection of the 5-mm pituitary microadenoma (arrow)

**Fig. 5:**
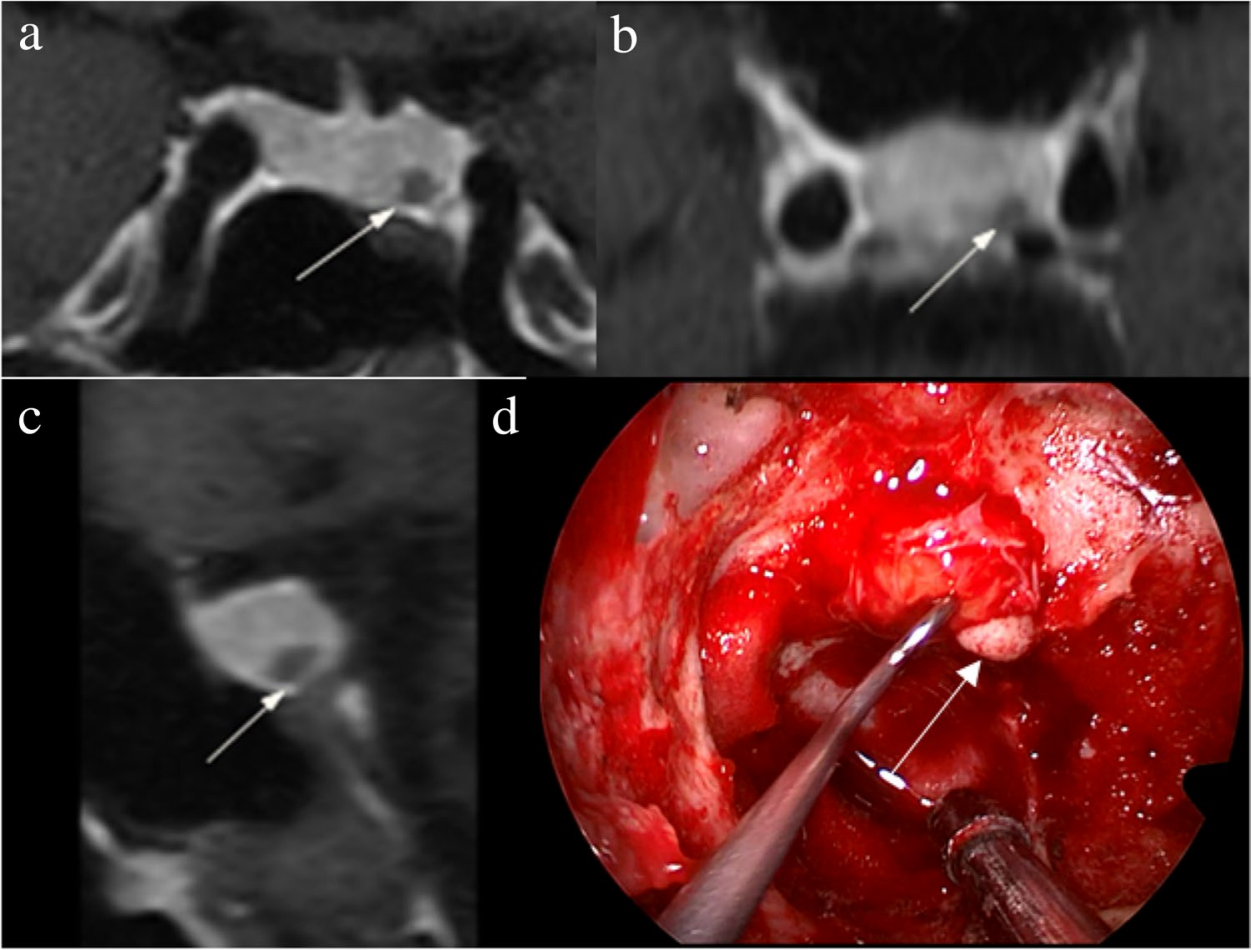
Images in a 48-year-old woman with Cushing’s disease. Preoperative high-resolution contrast-enhanced MRI using three-dimensional fast spin echo sequence shows a 4-mm pituitary microadenoma (arrow) with delayed enhancement is well delineated on the left side of the pituitary gland on (**a**) coronal, (**b**) reconstructed axial, and (**c**) reconstructed sagittal contrast-enhanced T1-weighted images. **d** Intraoperative endoscopic photograph during transsphenoidal surgery after exposure of the sellar floor shows a round pituitary microadenoma (arrow)

Figure 2 shows that a 5-mm pituitary microadenoma was identified on preoperative pituitary MRI. The margin of the lesion was fully delineated on hrMRI, but not on cMRI and dMRI. Figure 3 shows that a 3-mm pituitary microadenoma was missed on cMRI, but identified on dMRI and hrMRI. Figure 4 shows that a 5-mm pituitary microadenoma was correctly diagnosed on hrMRI, but missed on cMRI or dMRI. Figure 5 shows that a 4-mm pituitary microadenoma was evident on coronal images as well as reconstructed axial and reconstructed sagittal images on hrMRI.

### Image quality of cMRI, dMRI, and hrMRI

Image quality scores of cMRI, dMRI, and hrMRI are presented in Table 3. Scores for overall image quality, sharpness, and structural conspicuity on hrMRI (overall image quality, 5.0 [IQR, 5.0–5.0]; sharpness, 5.0 [IQR, 4.5–5.0]; structural conspicuity, 5.0 [IQR, 5.0–5.0]) were higher than those on cMRI (overall image quality, 4.0 [IQR, 3.5–4.0]; sharpness, 4.0 [IQR, 3.0–4.0]; structural conspicuity, 4.0 [IQR, 4.0–4.0]; *p* < 0.001 for all) and dMRI (overall image quality, 4.0 [IQR, 4.0–4.0]; sharpness, 4.0 [IQR, 4.0–4.0]; structural conspicuity, 4.0 [IQR, 4.0–4.5]; *p* < 0.001 for all).

**Table 3:** Image quality scores on cMRI, dMRI, and hrMRI

	cMRI	*p* value*	hrMRI	*p* value^†^	dMRI
Overall image quality	4.0 (3.5–4.0)	< .001	5.0 (5.0–5.0)	< .001	4.0 (4.0–4.0)
Sharpness	4.0 (3.0–4.0)	< .001	5.0 (4.5–5.0)	< .001	4.0 (4.0–4.0)
Structural conspicuity	4.0 (4.0–4.0)	< .001	5.0 (5.0–5.0)	< .001	4.0 (4.0–4.5)

Note: data are medians with interquartile ranges in parentheses. Abbreviations: *cMRI*, conventional contrast-enhanced MRI; *dMRI*, dynamic enhanced MRI; *hrMRI*, high-resolution contrast-enhanced MRI

^*^p value for comparison between cMRI and hrMRI

^†^*p* value for comparison between dMRI and hrMRI

The SNR and CNR measurements are shown in Table 4. The SNR of the pituitary microadenomas on hrMRI (67.5 [IQR, 51.2–92.1]) was lower than that on cMRI (82.3 [IQR, 61.8–127.2], *p* < 0.001), but higher than that on dMRI (53.9 [IQR, 35.2–72.6], *p* = 0.001). The CNR on hrMRI (26.2 [IQR, 15.1–41.0]) was higher than that on cMRI (10.6 [IQR, 0–42.6], *p* = 0.023) and dMRI (11.2 [IQR, 0–29.8], *p* < 0.001).

**Table 4:** SNR and CNR on cMRI, dMRI, and hrMRI

	cMRI	*p* value*	hrMRI	*p* value^†^	dMRI
SNR	82.3 (61.8–127.2)	< .001	67.5 (51.2–92.1)	.001	53.9 (35.2–72.6)
CNR	10.6 (0–42.6)	.023	26.2 (15.1–41.0)	< .001	11.2 (0–29.8)

Data are medians with interquartile ranges in parentheses. Abbreviations: *cMRI*, conventional contrast-enhanced MRI; *CNR*, contrast-to-noise ratio; *dMRI*, dynamic enhanced MRI; *hrMRI*, high-resolution contrast-enhanced MRI; *SNR*, signal-to-noise ratio

^*^p value for comparison between cMRI and hrMRI

^†^*p* value for comparison between dMRI and hrMRI

## Discussion

The identification of pituitary microadenomas is considerably challenging but critical in patients with ACTH-dependent Cushing’s syndrome. Our study demonstrated that hrMRI with 3D FSE sequence had higher diagnostic performance (AUC, 0.95–0.97) than cMRI (AUC, 0.74–0.75; *p* ≤ 0.002) and dMRI (AUC, 0.59–0.68; *p* ≤ 0.001) for identifying pituitary microadenomas. To our knowledge, there are no previous studies specifically evaluating the identification of pituitary microadenomas on hrMRI with 3D FSE sequence by comparison with cMRI and dMRI in patients with ACTH-dependent Cushing’s syndrome, and this is the largest study conducted in ACTH-secreting microadenomas with a sensitivity of more than 90%.

Recently, techniques for pituitary evaluation have developed rapidly. Because of false negatives and false positives on cMRI and dMRI using 2D FSE sequence [7, 9, 10], a 3D SPGR sequence was introduced for identifying pituitary adenomas. Previous studies demonstrated that the 3D SPGR sequence performed better than the 2D FSE sequence in the identification of pituitary adenomas with a sensitivity of up to 80% [11–13]. In patients with hyperprolactinemia, the 3D FSE sequence was recommended [14] and the 3D FSE sequence has rapidly developed recently with superior image quality [15, 16], suggesting that the 3D FSE sequence may be a reliable alternative for identifying pituitary adenomas. However, to our knowledge, few studies have investigated the diagnostic performance of the 3D FSE sequence for identifying ACTH-secreting pituitary adenomas. To fill the gaps, we conducted the current study and revealed that images obtained with the 3D FSE sequence had higher sensitivity (90–93%) in identifying pituitary microadenomas, than that in previous studies using the 3D SPGR sequence [8, 11–13].

There is a trade-off between spatial resolution and image noise. The reduced slice thickness can overcome the partial volume averaging effect, but it is associated with increased image noise [17]. Strikingly, our study showed that hrMRI had higher image quality scores than cMRI and dMRI, in terms of overall image quality, sharpness, and structural conspicuity. The SNR of the pituitary microadenomas on cMRI was slightly higher than that on hrMRI in our study. This is because the SNR was calculated as the mean signal intensity of the pituitary gland (instead of the pituitary microadenoma) divided by noise when no microadenoma was identified, and the mean signal intensity of the pituitary gland is higher than that of the pituitary microadenoma. About 40% of pituitary microadenomas were missed on cMRI, whereas less than 10% of pituitary microadenomas were missed on hrMRI. Given the situation mentioned above, the SNR on hrMRI was lower than that on cMRI. However, the CNR on hrMRI was significantly higher than that on cMRI and dMRI. Therefore, hrMRI in our study can dramatically improve the spatial resolution with high CNR, enabling the better identification of pituitary microadenomas.

The identification of pituitary adenomas on preoperative MRI in patients with ACTH-dependent Cushing’s syndrome could help the differential diagnosis of Cushing’s syndrome and aids surgical resection of lesions. It should be noted that most of the pituitary adenomas in patients with Cushing’s disease are microadenomas [5, 6]. In our study, all the tumors are microadenomas with a median diameter of 5 mm (IQR, 4–5 mm), making the diagnosis more challenging. The sensitivity of identifying pituitary adenomas decreased from 80 to 72% after excluding macroadenomas in a previous study [12], whereas the sensitivity of identifying pituitary microadenomas in our study was 90–93% on hrMRI. In the current study, hrMRI performed better than cMRI, dMRI, and combined cMRI and dMRI, with high AUC (0.95–0.97), high sensitivity (90–93%), and high specificity (100%), superior to previous studies [8, 11–13]. The high sensitivity of hrMRI for identifying pituitary adenomas will help surgeons improve the postoperative remission rate [4]. The high specificity of hrMRI will assist clinicians to consider ectopic ACTH syndrome, and then perform imaging to identify ectopic tumors. Besides, the inter-observer agreement for identifying pituitary microadenomas was almost perfect on hrMRI (*κ* = 0.91), which was moderate on cMRI (*κ* = 0.50) and dMRI (*κ* = 0.57). Therefore, hrMRI using the 3D FSE sequence is a potential alternative that can significantly improve the identification of pituitary microadenomas.

Limitations of the study included its retrospective nature and the relatively small sample size in patients with ectopic ACTH syndrome as negative controls. The bias may be introduced in the patient inclusion process. Only those patients who underwent all the cMRI, dMRI, and hrMRI scans were included. In fact, some patients will bypass hrMRI when obvious pituitary adenomas were detected on cMRI and dMRI. These patients were not included in the current study because of lack of hrMRI findings. Given the situation, the sensitivity of identifying pituitary adenomas will be higher with the enrollment of these patients. Besides, the timing of the sequence acquisition after contrast injection is essential [16] and bias may be introduced due to the postcontrast enhancement curve of both the pituitary gland and the microadenoma [14]. In the future, a prospective study with different sequence acquisition orders is needed to minimize possible interference caused by the postcontrast enhancement curve. Moreover, a larger sample size is also needed to verify the diagnostic performance of hrMRI using 3D FSE sequence for identifying pituitary microadenomas and to determine whether it can replace 2D FSE or 3D SPGR sequences for routinely evaluating the pituitary gland.

In conclusion, hrMRI with 3D FSE sequence showed higher diagnostic performance than cMRI and dMRI for identifying pituitary microadenomas in patients with Cushing’s syndrome.

## Supplementary Information

Below is the link to the electronic supplementary material.

Supplementary file1 (PDF 242 kb)

## Abbreviations

ACTH: Adrenocorticotropic hormone
AUC: Area under the receiver operating characteristics curve
cMRI: Conventional contrast-enhanced MRI
CNR: Contrast-to-noise ratio
dMRI: Dynamic contrast-enhanced MRI
FSE: Fast spin echo
hrMRI: High-resolution contrast-enhanced MRI
IQR: Interquartile range
SNR: Signal-to-noise ratio
SPGR: Spoiled gradient recalled

## Declarations

### Guarantor

The scientific guarantor of this publication is Feng Feng.

### Statistics and biometry

No complex statistical methods were necessary for this paper.

### Methodology

• retrospective

• diagnostic or prognostic study

• performed at one institution
