# Supplementary material for: High-resolution contrast-enhanced MRI with three-dimensional fast spin echo improved the diagnostic performance for identifying pituitary microadenomas in Cushing’s syndrome
Source: Eur Radiol. 2023 May 22;33(9):5984–92. doi: 10.1007/s00330-023-09585-1 (PMC10415427; doi:10.1007/s00330-023-09585-1)
Supplement: Supplementary file 1 — Supplementary file1 (PDF 242 kb) [file 330_2023_9585_MOESM1_ESM.pdf]

Supplementary material

Table S1: MR Acquisition Parameters

Sequence	FOV (cm^2)	Slice thickness/spacing (mm)	TR/TE (msec)	Acceleration factor	Number of slices	NEX	Matrix	Scan time
Coronal 2D FSE T2WI	20 × 20	4/1	4100/90	/	20	1.2	320 × 320	49s
Coronal 2D FSE T1WI	18 × 16.2	3/0.6	400/12	/	8	2	256 × 192	49s
Sagittal fs 3D FSE T1WI	16.5 × 16.5	3/0	460/16	2	16	2	256 × 224	60s
Dynamic CE coronal 2D FSE T1WI	19 × 17.1	2/0.5	375/14	/	6/phase × 6 phases	1	288 × 192	23s/phase × 6 phases
CE coronal 2D FSE T1WI	18 × 16.2	3/0.6	400/12	/	8	2	256 × 192	49s
CE sagittal fs 3D FSE T1WI	16.5 × 16.5	3/0	460/16	2	16	2	256 × 224	60s
CE coronal fs 3D FSE T1WI	15.2 × 15.2	1.2/-0.6	390/15	1.25	32	6	256 × 256	4min 30s

Abbreviations: CE, contrast-enhanced; FOV, field of view; fs, fat-saturated; FSE, fast spin-echo; NEX, number of excitation; T1WI, T1-weighted imaging; T2WI, T2-weighted imaging; TR/TE, repetition time/echo time.

Table S2: An example for image quality assessment

	Figure 4a (cMRI)	Figure 4b (dMRI)	Figure 4c (hrMRI)
Overall image quality	4	3	5
Sharpness	4	3	5
Structural conspicuity	4	4	5

Abbreviations: cMRI, conventional contrast-enhanced MRI; dMRI, dynamic enhanced MRI; hrMRI, high-resolution contrast-enhanced MRI.

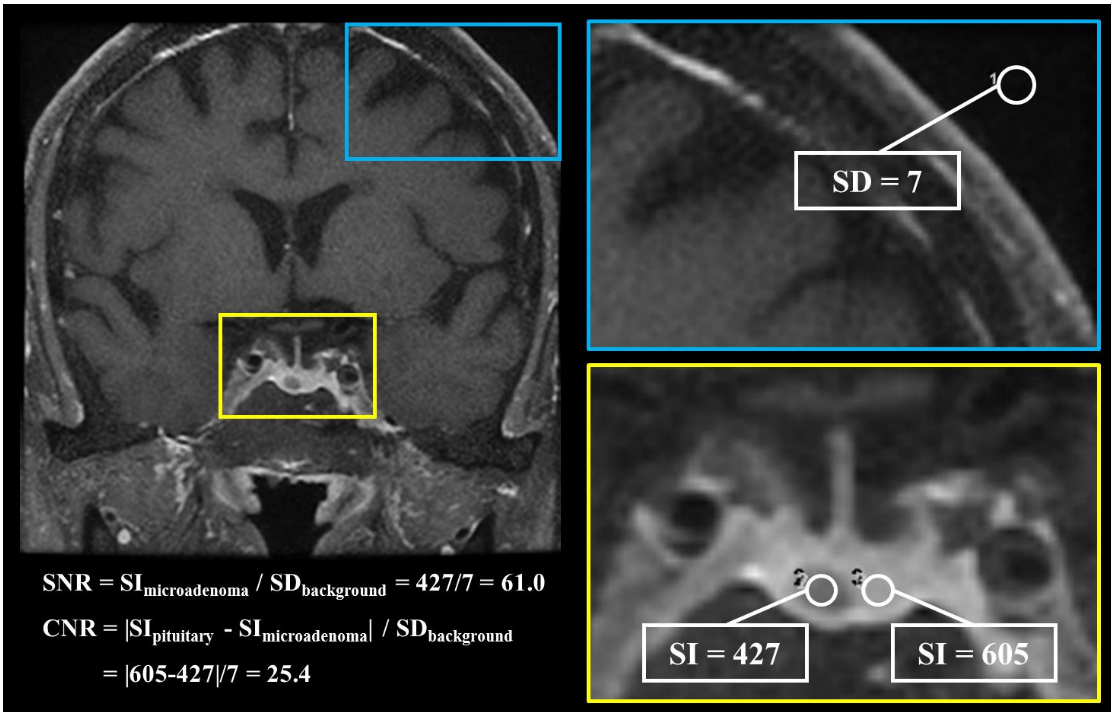

Supplementary Figure 1: Exemplary ROI placement in a contrast-enhance pituitary MRI to compute the SNR and CNR. CNR = contrast-to-noise ratio, SD = standard deviation, SI = signal intensity, SNR = signal-to-noise ratio.

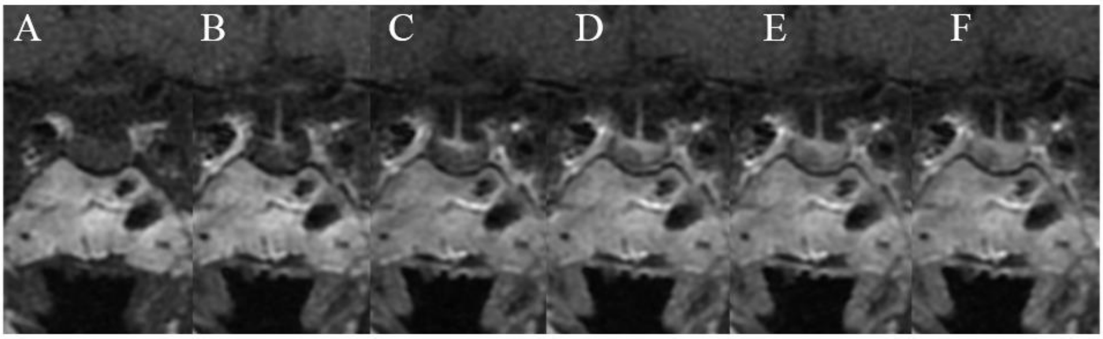

Supplementary Figure 2: Images of different phases in a 56-year-old man with Cushing's disease on dMRI. The pituitary microadenoma can be identified on (d), (e) and (f), and (d) was chosen as the "best image" for the SNR and CNR calculation. CNR = contrast-to-noise ratio, dMRI = dynamic contrast-enhanced MRI, SNR = signal-to-noise ratio.
